# YM155 sensitizes TRAIL-induced apoptosis through cathepsin S-dependent down-regulation of Mcl-1 and NF-κB-mediated down-regulation of c-FLIP expression in human renal carcinoma Caki cells

**DOI:** 10.18632/oncotarget.11137

**Published:** 2016-08-09

**Authors:** Seon Min Woo, Kyoung-jin Min, Bo Ram Seo, Taeg Kyu Kwon

**Affiliations:** ^1^ Department of Immunology, School of Medicine, Keimyung University, Dalseo-Gu, Daegu 704-701, South Korea

**Keywords:** YM155, TRAIL, Mcl-1, c-FLIP, lysosome, NF-κB

## Abstract

YM155, a small-molecule survivin inhibitor, has been reported for its anti-cancer activity in various cancer cells. In this study, we investigated the effect of YM155 to enhance TRAIL-mediated apoptosis in human renal carcinoma cells. We found that YM155 alone had no effect on apoptosis, however, combined treatment with YM155 and TRAIL markedly induced apoptosis in human renal carcinoma cells (Caki, ACHN, and A498), breast cancer cells (MDA-MB231), and glioma cells (U251MG), but not normal cells [mesangial cell (MC) and human skin fibroblast (HSF)]. YM155 induced down-regulation of Mcl-1 expression at the post-translational levels, and the overexpression of Mcl-1 markedly inhibited YM155 plus TRAIL-induced apoptosis. Furthermore, YM155 induced down-regulation of c-FLIP mRNA expression through inhibition of NF-κB transcriptional activity. Ectopic expression of c-FLIP markedly blocked YM155-induced TRAIL sensitization. Taken together, our results suggested that YM155 sensitizes TRAIL-mediated apoptosis via down-regulation of Mcl-1 and c-FLIP expression in renal carcinoma Caki cells.

## INTRODUCTION

Tumor necrosis factor (TNF)-related apoptosis-inducing ligand (TRAIL) selectively induces apoptotic cell death in cancer cells but has no effect on normal cells, therefore, it has obtained interest as a promising agent for cancer therapy [1]. TRAIL induced apoptosis begins at the point when it interacts to death receptor (DR) 4 and DR5, and forms death-inducing signal complex (DISC) with recruitment of FAS-associated protein with death domain (FADD) and caspase-8. Activation of caspase-8 by DISC activates caspase-3, and consequently induces apoptosis [2]. However, many tumor cells have resistance to TRAIL-mediated apoptosis [3]. The underlying mechanisms of TRAIL resistance are associated with down-regulation of DR expression, up-regulation of anti-apoptotic proteins expression, such as c-FLIP, anti-apoptotic Bcl-2 family (Bcl-2, Bcl-xL and Mcl-1), inhibitor of apoptosis proteins (IAPs), and down-regulation of pro-apoptotic Bcl-2 family proteins (Bax, Bim and PUMA) [4–8]. Therefore, the combined treatment with the TRAIL sensitizers is required to overcome TRAIL resistance.

Survivin is the smallest protein of the IAP gene family, which contains a single baculovirus IAP repeat (BIR) domain along with a long C-terminal α-helix [9]. It interferes with apoptosis by inhibiting the activation of caspase [10]. Moreover, survivin is overexpressed in malignant cells, including osteosarcoma [11], breast cancer [12], pancreatic cancer [13] and non-small cell lung cancer [14], and overexpression of survivin is associated with cancer cell survival and drug resistance [15–17]. YM155, a novel small-molecule survivin suppressant, has recently been reported to suppress survivin expression through inhibition of Sp1 [18] or ILF3/p54 complex [19] binding to the survivin promoter. Although YM-155 has been known as a survivin inhibitor, it also targets other apoptosis-related proteins. In prostate cancer cells, YM155 decreases survivin expression and induces autophagy-dependent apoptosis [20]. In addition, YM155 down-regulates survivin and Mcl-1 expression, leading to induction of caspase-8-dependent apoptosis in leukemia cells [21]. Moreover, YM155 induces caspase-independent cell death through PARP hyper-activation and AIF translocation from the cytosol to the nucleus in esophageal squamous-cell carcinoma xenograft model [22]. Anti-cancer effects of YM155 are promoted by its combined treatment with other drugs. For example, YM155 overcomes cisplatin resistance in head and neck cancer via reduction of cytoplasmic survivin levels [23]. YM155 increases the sensitivity to radiation through up-regulation of histone H2AX phosphorylation and activation of caspase-3 in non-small cell lung cancer cell lines [24]. In addition, YM155 enhances TRAIL-induced apoptosis through down-regulation of Mcl-1 in TRAIL-resistant glioma cells [25], and p38 MAPK-and CHOP-mediated DR5 up-regulation in breast cancer [26], respectively. However, the effect of YM155 on TRAIL sensitization in human renal carcinoma has not yet been investigated.

In this study, we investigated effect of YM155 on TRAIL-mediated apoptosis and molecular mechanisms of TRAIL sensitization in human renal carcinoma Caki cells. We found that YM155 sensitizes TRAIL-induced apoptosis through down-regulation of Mcl-1 by cathepsin S and c-FLIP expression through inhibition of NF-κB transcriptional activity. These results suggest that YM155 could be an effective sensitizer against TRAIL.

## RESULTS

### YM155 sensitizes Caki cells to TRAIL-mediated apoptosis

**Figure 1:**
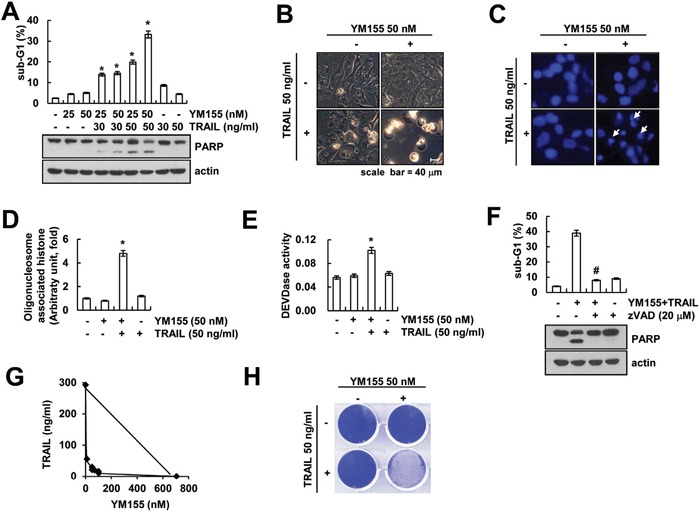
YM155 sensitizes Caki cells to TRAIL-mediated apoptosis **A.** Caki cells were treated with the indicated concentrations with TRAIL in the presence or absence of the indicated concentrations of YM155 for 24 h. The sub-G1 fraction was measured by flow cytometry as an indicator of the level of apoptosis. The protein expression levels of PARP and actin were determined by western blotting. The level of actin was used as a loading control. **B-E.** Caki cells were treated with 50 ng/ml TRAIL in the presence or absence of 50 nM YM155 for 24 h. The cell morphology was examined using interference light microscopy (B). The condensation and fragmentation of the nuclei were detected by 4′,6′-diamidino-2-phenylindole staining (C). The cytoplasmic histone-associated DNA fragments were determined by a DNA fragmentation detection kit (D). Caspase activities were determined with colorimetric assays using caspase-3 (DEVDase) assay kits (E). **F.** Caki cells were treated with 50 nM YM155 plus 50 ng/ml TRAIL for 24 h in the presence or absence of 20 μM z-VAD-fmk (z-VAD). The sub-G1 fraction was measured by flow cytometry. The protein expression levels of PARP and actin were determined by western blotting. The level of actin was used as a loading control. **G.** Isoboles were obtained by plotting the combined concentrations of each drug required to produce 50% cell death. The straight line connecting the IC_50_ values obtained for the two agents when applied alone corresponded to the addition of their independent effects. Values below this line indicate synergy, whereas values above this line indicate antagonism. **H.** Effect of combined treatment with YM155 and TRAIL on long-term survival. Caki cells were treated with 50 ng/ml TRAIL in the presence or absence of 50 nM YM155. Clonogenic survival was determined by staining colonies with crystal violet and visualized with 0.4% coomassie blue by a digital camera. The values in panel (A, D, E and F) represent the mean ± SD from three independent samples. * *p* < 0.05 compared to the control. # *p* < 0.01 compared to the combined treatment with YM155 and TRAIL.

Because YM155 has anti-cancer effect on various types of cancer cell [21, 27, 28], we investigated whether YM155 could sensitize TRAIL-mediated apoptosis in human renal carcinoma Caki cells. As shown in Figure 1A, when Caki cells were treated with YM155 alone or TRAIL alone, cell death was not induced. However, combined treatment with YM155 and TRAIL markedly induced sub-G1 population and PARP cleavage in a dose-dependent manner (Figure 1A). Furthermore, combined treatment with YM155 plus TRAIL exhibited the typical apoptotic morphologies, chromatin condensation in the nuclei and DNA fragmentation (Figure 1B-1D). We next examined whether combined treatment with YM155 and TRAIL induces caspase-dependent apoptosis. Combined treatment with YM155 plus TRAIL increased the caspase-3 activity (Figure 1E), but not caspase-2, -8 and -9 (Supplementary Figure S1). To confirm whether the YM155 plus TRAIL-mediated apoptosis is involved in activation of caspase, we examined the effect of pretreatment with z-VAD-fmk, a pan-caspase inhibitor. The z-VAD markedly inhibited co-treatment with YM155 and TRAIL-induced apoptosis and PARP cleavage (Figure 1F). Moreover, we investigated whether combined treatment with YM155 and TRAIL have synergistic effects. Combined treatment with various concentrations of YM155 and TRAIL showed synergistic effects (Figure 1G), and also inhibited colony formation (Figure 1H). These data indicated that YM155 enhances TRAIL-mediated apoptosis via caspase activation in human renal carcinoma Caki cells.

### YM155 decreases the mitochondrial membrane potential (MMP)

**Figure 2:**
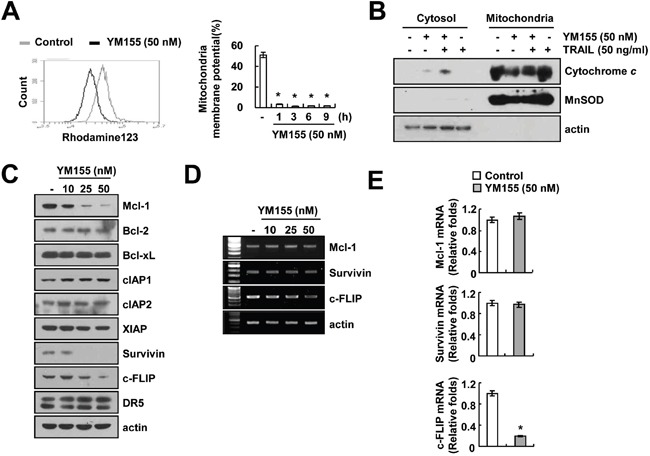
YM155 induces loss of mitochondrial membrane potential (MMP) **A.** Caki cells were treated with 50 nM YM155 for 3 h (left panel) or the indicated time periods (right panel) and loaded with a rhodamine123 fluorescent dye. The mitochondrial membrane potential (MMP) was measured using a flow cytometer. **B.** Caki cells were treated with 50 ng/ml TRAIL in the presence or the absence of 50 nM YM155 for 24 h. Cytoplasmic fractions were analyzed for cytochrome *c* release. The level of MnSOD was used as a mitochondria loading control. The level of actin was used as a loading control. **C-E.** Caki cells were treated with the indicated concentrations of YM155 for 24 h. The protein levels of Mcl-1, Bcl-2, Bcl-xL, cIAP1, cIAP2, XIAP, survivin, c-FLIP and DR5 were determined by western blotting (C). The mRNA levels of Mcl-1, survivin and c-FLIP were determined by RT-PCR (D) and quantitative PCR (E), respectively. The level of actin was used as the loading control. The values in panel (A and E) represent the mean ± SD from three independent samples. * *p* < 0.05 compared to the control.

The loss of mitochondrial membrane potential (MMP) and cytochrome *c* release are critical events of mitochondria-mediated apoptosis [29]. Therefore, we examined the association of YM155 and TRAIL combination with the loss of MMP, by using rhodamine123 fluorescence dye and found that, YM155 markedly reduced the MMP levels (Figure 2A). Release of cytochrome *c* from mitochondria to cytosol was also observed in combined treatment with YM155 plus TRAIL (Figure 2B). Next, we investigated the potential of YM155 to regulate the expression levels of apoptosis-related proteins and we observed that YM155 efficiently down-regulated the expression of Mcl-1, survivin and c-FLIP proteins in a dose-dependent manner. In contrast, levels of Bcl-2, Bcl-xL, cIAP1, cIAP2, XIAP and DR5 were not altered in response to YM155 (Figure 2C). We analyzed the surface expression of DR5 receptor by flow cytometry. YM155 did not change DR5 expression on cell surface (Supplementary Figure S2). Furthermore, we examined the effect of YM155 in modulation of Mcl-1, survivin and c-FLIP expression at the transcriptional levels. As shown in Figure 2D and 2E, YM155 induced down-regulation of c-FLIP mRNA expression, but not Mcl-1 and survivin. These results indicated that YM155 induced down-regulation of Mcl-1 and survivin expression at the post-transcriptional levels and c-FLIP expression at the transcriptional levels.

### Mcl-1 down-regulation by YM155 contributes to the sensitization of TRAIL-mediated apoptosis

**Figure 3:**
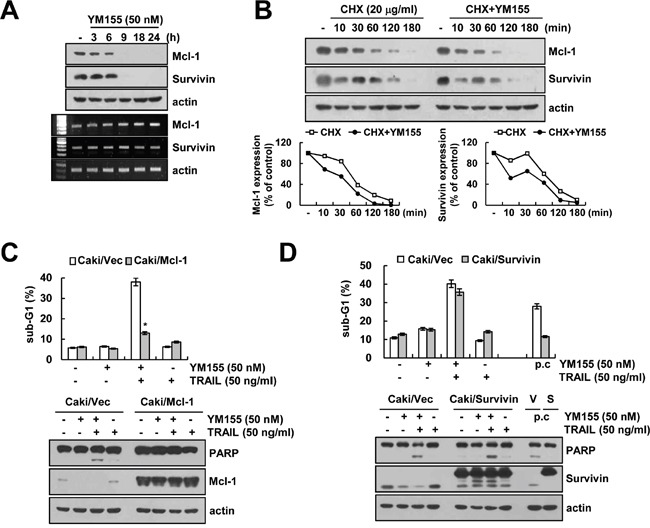
Down-regulation of Mcl-1 by YM155 is associated with the induction of TRAIL-mediated apoptosis **A.** Caki cells were treated with 50 nM YM155 for the indicated time periods. The protein and mRNA expression levels of Mcl-1, survivin and actin were determined by western blotting and RT-PCR, respectively. The level of actin was used as a loading control. **B.** Caki cells were treated with or without 50 nM YM155 in the presence of 20 μg/ml cyclohexamide (CHX) for the indicated time periods. The protein expression levels of Mcl-1, survivin and actin protein levels were determined by western blotting. The level of actin was used as a loading control. The band intensity of the Mcl-1 and survivin protein was measured using ImageJ (public domain JAVA image-processing program; http://rsb.info.nih.gov/ij, lower panel). **C.** Vector-transfected cells (Caki/Vec) and Mcl-1-overexpressing cells (Caki/Mcl-1) were treated with 50 ng/ml TRAIL in the presence or absence of 50 nM YM155 for 24 h. The level of apoptosis was analyzed by the sub-G1 fraction using flow cytometry (upper panel). The protein expression levels of PARP, Mcl-1 and actin were determined by western blotting. The level of actin was used as a loading control. **D.** Vector-transfected cells (Caki/Vec) and survivin-overexpressing cells (Caki/Survivin) were treated with 50 ng/ml TRAIL in the presence or absence of 50 nM YM155 for 24 h. The level of apoptosis was analyzed by the sub-G1 fraction using flow cytometry (upper panel). The protein expression levels of PARP, survivin and actin were determined by western blotting. The level of actin was used as a loading control (lower panel). The values in panel (C and D) represent the mean ± SD from three independent samples. *p < 0.01 compared to the YM155 plus TRAIL treated Caki/Vec.

Next, we investigated whether YM155 could modulate protein stability of Mcl-1 and survivin. We first determined the time-dependent effect of YM155 in down-regulation of Mcl-1 and survivin protein expression. From the results, we observed that YM155 downregulated the expression of Mcl-1 and survivin within 6 and 9 h. However, Mcl-1 and survivin mRNA expression was not changed by YM155 treatment (Figure 3A). Next, Caki cells were pretreated with cycloheximide (CHX), an inhibitor of protein biosynthesis, followed by treatment with YM155 for up to 180 min. CHX alone gradually reduced Mcl-1 and survivin expression, but combined treatment with CHX and YM155 more rapidly reduced both proteins expression (Figure 3B). To examine the importance of Mcl-1 and survivin down-regulation in YM155 plus TRAIL-induced apoptosis, we used Mcl-1 and survivin-overexpressing Caki cells. The induction of apoptosis and PARP cleavage by combined treatment with YM155 and TRAIL markedly blocked in Mcl-1-overexpressing cells (Figure 3C). However, combined treatment with YM155 and TRAIL was markedly increased sub-G1 population and PARP cleavage in survivin-overexpressing cells compared with vector cells (Figure 3C), even though apoptosis by positive control (galangin plus TRAIL) was reduced in survivin-overexpressing cells [30]. These data suggest that the down-regulation of Mcl-1 expression has a critical role on YM155-medated TRAIL sensitization, rather than survivin.

### YM155 induces cathepsin S-mediated down-regulation of Mcl-1 expression

**Figure 4:**
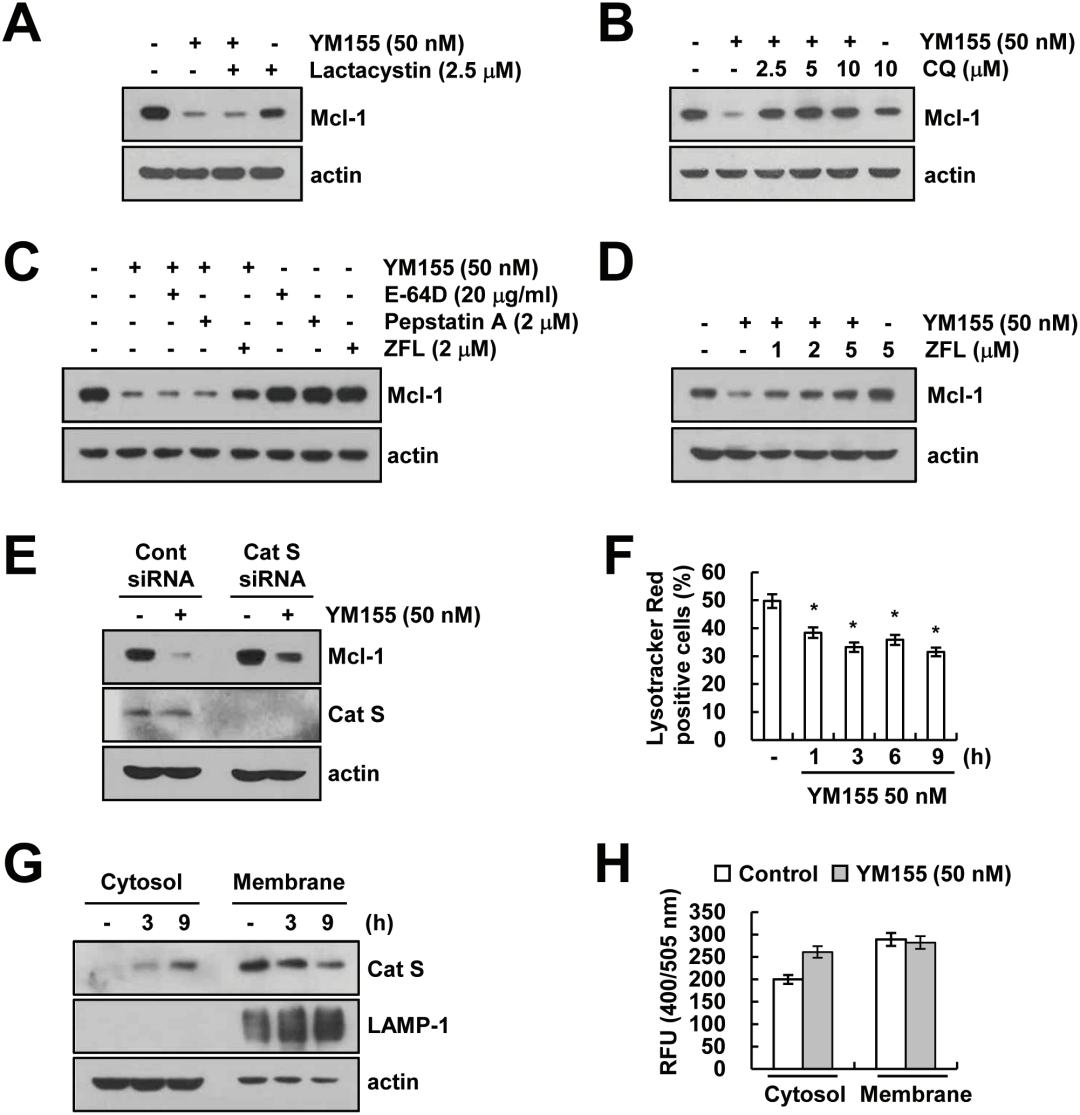
YM155 induces down-regulation of Mcl-1 expression in a lysosomal cathepsin S-dependent manner **A.** Caki cells were pretreated with 2.5 μM lactacystin for 30 min, and then treated with 50 nM YM155 for 24 h. The protein expression levels of Mcl-1 and actin were determined by western blotting. The level of actin expression was used as a loading control. **B.** Caki cells were pretreated with the indicated concentrations with chloroquine (CQ) for 30 min, and then treated with 50 nM YM155 for 24 h. The protein expression levels of Mcl-1 and actin were determined by western blotting. The level of actin expression was used as a loading control. **C.** Caki cells were pretreated with 20 μg/ml E-64D, 2 μM pepstatin A and 2 μM ZFL for 30 min, and then treated with 50 nM YM155 for 24 h. The protein expression levels of Mcl-1 and actin were determined by western blotting. The level of actin expression was used as a loading control. **D.** Caki cells were pretreated with the indicated concentrations with ZFL for 30 min, and then treated with 50 nM YM155 for 24 h. The protein expression levels of Mcl-1 and actin were determined by western blotting. **E.** Caki cells were transiently transfected control siRNA (Cont siRNA) or cathepsin S siRNA (Cat S siRNA). Twenty-four hours after transfection, cells were treated with 50 nM YM155 for 24 h. The protein expression levels of Mcl-1, cathepsin S and actin were determined by western blotting. The level of actin expression was used as a loading control. **F.** Caki cells were treated with 50 nM YM155 for the indicated time periods and then loaded with Lysotracker fluorescent dye. The fluorescence intensity was detected by fluorescence microscopy. **G.** Caki cells were treated with 50 nM YM155 for the indicated time periods. After treatment, cytosol and membrane fractions (lysosome rich fraction) were prepared and the protein expression levels of cathepsin S, LAMP-1 and actin were determined by western blotting. The level of actin expression was used as a loading control. **H.** The cathepsin S enzyme activity was determined by a cathepsin S activity assay kit. The values in panel (F) represent the mean ± SD from three independent samples. * *p* < 0.05 compared to the control.

Since, degradation of Mcl-1 is mainly regulated by the ubiquitin-proteasome system [31, 32], we explored whether proteasome inhibitor (lactacystin) reverses YM155-mediated down-regulation of Mcl-1 expression. However, we found that lactacystin had no effect on Mcl-1 down-regulation by YM155 treatment (Figure 4A). Since lysosomes have a role in intracellular protein degradation and recycling [33], we investigate that down-regulation of Mcl-1 expression by YM155 is associated with lysosomal degradation pathway or not. Pretreatment of cells with lysosomal inhibitor [chloroquine (CQ)] inhibited Mcl-1 down-regulation by YM155 (Figure 4B). Cathepsins are the most abundant lysosomal enzymes, including cysteine protease, aspartic protease and serine protease [34]. Therefore, we examined the effect of pretreatment with cathepsin inhibitors [E-64D, pepstatin A and Z-FL-COCHO (ZFL)] on Mcl-1 down-regulation in YM155-treated cells. Interestingly, pretreatment with ZFL, a specific inhibitor of cathepsin S, restored YM155-induced down-regulation of Mcl-1 expression (Figure 4C and 4D). However, an inhibitor of cathepsin B (E-64D) or cathepsin D (pepstatin A) did not modulate Mcl-1 expression decreased by YM155 (Figure 4C). Furthermore, down-regulation of cathepsin S by siRNA reversed YM155-induced down-regulation of Mcl-1 expression (Figure 4E). However, knock-down of cathepsin B and cathepsin D by siRNA did not alter Mcl-1 expression in YM155-treated cells (Supplementary Figure S3). Next, we investigated the effect of YM155 on lysosomal membrane permeabilization (LMP) and found that YM155 induced loss of lysosomal membrane integrity (Figure 4F), and released cathepsin S into cytosol from lysosome within 3 h (Figure 4G). Moreover, YM155 increased catehpsin S enzyme activity in cytosol (Figure 4H). These data indicated that YM155 induced down-regulation of Mcl-1 expression via lysosomal release of cathepsin S.

### Down-regulation of c-FLIP is associated with YM155 plus TRAIL-mediated apoptosis

**Figure 5:**
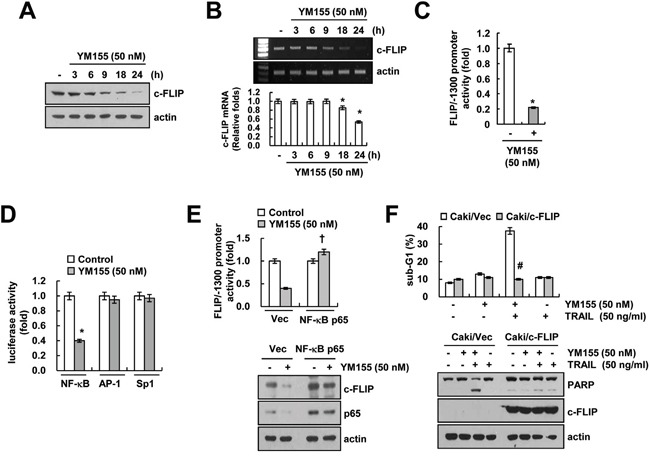
Down-regulation of c-FLIP by inhibition of NF-κB is involved in YM155 plus TRAIL-mediated apoptosis **A** and **B.** Caki cells were treated with 50 nM YM155 for the indicated time periods. The protein levels of c-FLIP and actin were determined by western blotting (A). The mRNA levels of c-FLIP and actin were determined by RT-PCR (B, upper panel) and quantitative PCR (B, lower panel). The level of actin was used as the loading control. **C.** Caki cells were transiently transfected with a plasmid harboring the luciferase gene under the control of the FLIP/−1300 promoter. After transfection, the Caki cells were treated with 50 nM YM155 for 24 h. After treatment, the cells were lysed, and the luciferase activity was analyzed. **D.** Caki cells were transiently transfected with NF-κB-, AP-1- and Sp1-luciferase construct. After transfection, the Caki cells were treated with 50 nM YM155 for 24 h. After treatment, the cells were lysed, and the luciferase activity was analyzed. **E.** Caki cells were transiently co-transfected with a plasmid harboring the luciferase gene under the control of the FLIP/−1300 promoter and vector or NF-κB subunit (p65). After transfection, the Caki cells were treated with 50 nM YM155 for 24 h. After treatment, the cells were lysed, and the luciferase activity was analyzed (upper panel). The protein expression levels of c-FLIP, p65 and actin were determined by western blotting. The level of actin expression was used as a loading control (lower panel). **F.** Vector-transfected cells (Caki/Vec) and c-FLIP-overexpressing cells (Caki/c-FLIP) were treated with 50 ng/ml TRAIL in the presence or absence of 50 nM YM155 for 24 h. The level of apoptosis was analyzed by the sub-G1 fraction using flow cytometry (upper panel). The protein expression levels of PARP, c-FLIP and actin were determined by western blotting. The level of actin was used as a loading control (lower panel). The values in panel (B, C, D, E and F) represent the mean ± SD from three independent samples. * *p* < 0.05 compared to the control. † *p* < 0.01 compared to the YM155-treated Caki/Vector. # *p* < 0.01 compared to the YM155 plus TRAIL treated Caki/Vector.

As shown in Figure 2D, YM155 induced down-regulation of c-FLIP at the transcriptional level. Next, we examined the mechanism involved in the modulation of c-FLIP expression. We observed that, YM155 induced down-regulation of c-FLIP protein (Figure 5A) and mRNA expression within 9 h (Figure 5B), along with marked inhibition of c-FLIP promoter activity as well (Figure 5C). It has been reported that the regulation of c-FLIP mRNA expression is mediated by NF-κB [35], activator protein 1 (AP-1) [36] and specificity protein 1 (Sp1)-associated transcriptional activity [36]. YM155 markedly inhibited NF-κB transcriptional activity, but had no effect on AP-1 and Sp1 transcriptional activity (Figure 5D). Subsequently, to further confirm the importance of NF-κB transcriptional activity on c-FLIP down-regulation, cells were transiently overexpressed with p65. Ectopic expression of p65 reversed YM155-induced inhibition of c-FLIP promoter activity and expression (Figure 5E). These data indicated that inhibition of NF-κB transcriptional activity is involved in YM155-induced down-regulation of c-FLIP expression. Next, we investigated the role of c-FLIP down-regulation in combined treatment with YM155 and TRAIL-mediated apoptosis. As shown in Figure 5F, overexpression of c-FLIP completely blocked apoptosis and PARP cleavage in YM155 plus TRAIL-treated cells (Figure 5F). These data indicated that down-regulation of c-FLIP by inhibition of NF-κB transcriptional activity plays a critical role on combined treatment with YM155 and TRAIL-induced apoptosis.

### Effect of YM155 plus TRAIL on apoptosis in other cancer cells and normal cells

**Figure 6:**
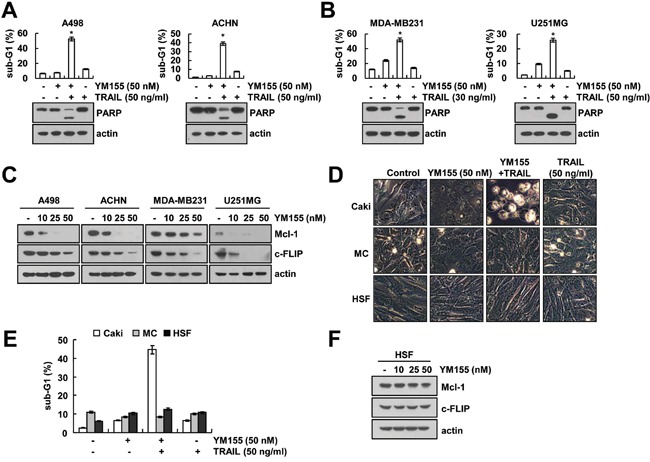
Effect of YM155 plus TRAIL treatment on apoptosis of other carcinoma cells and normal cells **A** and **B.** Renal carcinoma (A498 and ACHN), breast carcinoma (MDA-MB231), and glioma (U251MG) cells were treated with 30 or 50 ng/ml TRAIL in the presence or absence of 50 nM YM155 for 24 h. The level of apoptosis was assessed by measuring the sub-G1 fraction using flow cytometry (upper panel). The protein levels of PARP and actin were determined by western blotting. The level of actin was used as the loading control (lower panel). **C.** Cancer cells were treated with the indicated concentrations of YM155 for 24 h. The protein levels of Mcl-1, c-FLIP and actin were determined by western blotting. The level of actin was used as the loading control. **D** and **E.** Caki, mesangial cells (MC) and HSF cells were treated with50 ng/ml TRAIL in the presence or absence of 50 nM YM155 for 24 h. The cell morphology was examined using interference light microscopy (D). The level of apoptosis was assessed by measuring the sub-G1 fraction using flow cytometry (E). **F.** HSF normal cells were treated with the indicated concentrations of YM155 for 24 h. The protein levels of Mcl-1, c-FLIP and actin were determined by western blotting. The level of actin was used as the loading control. The values in panel (A, B and E) represent the mean ± SD from three independent samples. * *p* < 0.05 compared to the control.

We further investigated whether combined treatment with YM155 and TRAIL induces apoptosis in other cancer cells. Combined treatment with YM155 and TRAIL induced apoptosis and PARP cleavage in renal carcinoma cells (A498 and ACHN), breast carcinoma cells (MDA-MB231) and giloma cells (U251MG) (Figure 6A and 6B). Furthermore, YM155 also induced down-regulation of Mcl-1 and c-FLIP expression in other cancer cells (Figure 6C). In contrast, combined treatment with YM155 and TRAIL had no effect on apoptosis in normal cells [human mesangial cells (MC) and human skin fibroblast cells (HSF)] (Figure 6D and 6E). In addition, YM155 treatment did not change Mcl-1 and c-FLIP expression in normal cells (Figure 6F). Therefore, our data suggested that YM155 could selectively increase TRAIL-induced apoptosis in cancer cells.

## DISCUSSION

In this study, we demonstrated that YM155 enhances TRAIL-mediated apoptosis in cancer cells, but not in normal cells. YM155 induced down-regulation of Mcl-1 expression through LMP-mediated cathepsin S release into cytosol. In addition, inhibition of c-FLIP expression by YM155 is associated with down-regulation of NF-κB transcriptional activity. Down-regulation of Mcl-1 and c-FLIP by YM155 is main mechanism of sensitization of TRIAL-induced apoptosis. These findings support that YM155 treatment could be an attractive therapeutic strategy for induction of sensitization to TRAIL-mediated apoptosis.

We, in our study explored that inhibition of Mcl-1 expression plays a critical role on YM155-induced TRAIL sensitization. Previous studies have reported that YM155 could modulate Mcl-1 expression [21, 37–40]. However, the mechanism of Mcl-1 down-regulation is different, depending on cell types. For examples, YM155 induces down-regulation of Mcl-1 at the transcriptional level [40] or at the post-translational levels [41], but the molecular mechanism of Mcl-1 down-regulation by YM155 is not elucidated well. Degradation of Mcl-1 has mainly been known to be regulated by proteasome [31]. Cysteine cathepsins cleave anit-apoptotic and pro-apoptotic proteins, including Bcl-2, Bcl-xL, Bak, Bim, and Mcl-1, depending on cell context [42]. In addition, degradation of Mcl-1 by cathepsin B plays a critical role on FTY720-induced leukemia apoptosis [43]. Sachita et al., reported that YM155 modulate Mcl-1 expression via lysosome-dependent protein degradation in human oral cancer cell lines [41]. In our study, YM155 induced lysosomal membrane permeabilization (LMP), and then released cathepsin S into cytosol from lysosome in human renal carcinoma cells (Figure 4F and 4G). Down-regulation of Mcl-1 is associated with cathepsin S release (Figure 4E). Therefore, our data indicated that YM155-mediated LMP is important to induce cathepsin S-dependent down-regulation of Mcl-1 expression.

YM155 has been known as a survivin suppressant, and inhibit survivin transcription via inhibition of Sp1 binding to the survivin promoter [18] or disruption of ILF3/p54 complex [19]. However, it is reported that YM155 could modulate survivin expression at the post-transcriptional level. Sachita et al. reported that YM155 inhibit survivin protein expression, but not mRNA in human oral cancer cells [41]. Proteasome inhibitor (MG132) reversed YM155-induced survivin down-regulation [41]. As shown in Figure 3A and 3B, YM155 also induced down-regulation of survivin expression at the post-transcriptional level in human renal Caki cells. Interestingly, we also found that down-regulation of survivin is regulated in a lysosome dependent manner, but not proteasome (data not shown). As shown in Figure 5D, YM155 had no effect on Sp1 transcriptional activity in human renal carcinoma Caki cells. Therefore, this might be a possible reason why YM155 had no effect on survivin mRNA expression. Furthermore, as shown in Figure 3C and 3D, overexpression of Mcl-1 inhibited YM155 plus TRAIL-induced apoptosis, whereas survivin overexpression did not alter suggesting that, most of the YM155 effects depend on inhibition of survivin expression. In a previous study, Wanger et al. reported that YM155 inhibited proliferation and induced apoptosis in multiple myeloma cells, and down-regulation of Mcl-1 by YM155 is more important on its anti-cancer effect, rather than survivin [39]. Our study also suggests that down-regulation of Mcl-1 has more critical roles on YM155-induced TRAIL sensitization in human renal Caki cells.

The generation of reactive oxygen species (ROS) plays a major role in TRAIL sensitization [44–46]. A previous study reported that YM155 increased ROS production in esophageal squamous-cell carcinoma [22]. We found that YM155 increased intracellular ROS generation (Supplementary Figure 4A). However, ROS scavengers [N-acetyl-L-cysteine (NAC), trolox and glutathione ethyl ester (GEE)] did not affect apoptosis and PARP cleavage resulting from the combined treatment with YM155 and TRAIL (Supplementary Figure 4B). These results indicated that effect of YM155 on TRAIL sensitization is independent of ROS production.

In conclusion, our results suggested that YM155 induces TRAIL-mediated apoptosis through cathepsin S-dependent down-regulation of Mcl-1 expression and down-regulation of c-FLIP expression via down-regulation of NF-κB transcriptional activity in human renal cancer cells.

## MATERIALS AND METHODS

### Cell culture and materials

Human renal carcinoma (Caki, ACHN, and A498), human breast carcinoma cells (MDA-MB231), and human glioma cells (U251MG) were obtained from the American Type Culture Collection (Manassas, VA). Primary culture of human mesangial cells (Cryo NHMC) were purchased from Clonetics (San Diego, CA). The human skin fibroblast (HSF) was a gift from Dr. T.J. Lee (Yeungnam University, Korea). All cells were cultured in Dulbecco's Modified Eagle's Medium containing 10% fetal bovine serum, 20 mM HEPES buffer, 100 U/ml penicillin, 100 μg/ml streptomycin, and 100 μg/ml gentamicin. The PCR primers were purchased from Macrogen (Seoul, Korea). YM155 was purchased from Selleckchem (Houston, TX), and recombinant human TRAIL, z-VAD-fmk and anti-survivin antibody (AF886) was purchased from R&D system (Minneapolis, MN). Lactacystin was obtained from Cayman (Ann Arbor, MI). Pepstatin A, Z-FL-COCHO and anti-MnSOD (06-984) antibody was obtained from EMD Millipore (Darmstadt, Germany). Anti-Mcl-1 (sc-819), anti-Bcl-2 (sc-783), anti-cIAP2 (sc-7944), anti-cathepsin S (sc-6505), anti-LAMP1 (sc-5570) and anti-p65 (sc-8008) antibodies were purchased from Santa Cruz Biotechnology (Dallas, TX). Anti-cytochrome *c* (556433) and anti-XIAP (610762) antibodies were purchased from BD Biosciences (San Jose, CA). Anti-PARP (#9542), anti-Bcl-xL (#2764), anti-DR5 (#8074), anti-cIAP1 (#4952) antibodies were purchased from Cell Signaling Technology (Beverly, MA). Anti-c-FLIP (ALX-804-961-0100) antibody was obtained from Enzo life science (Farmington, NY). Anti-actin (A5441) antibody and other chemicals were purchased from Sigma Chemical Co. (St. Louis, MO).

### Flow cytometry analysis

For flow cytometry, the cells were resuspended in 100 μl of phosphate-buffered saline (PBS), and 200 μl of 95% ethanol was added while the cells were being vortexed. Then, the cells were incubated at 4°C for 1 h, washed with PBS, resuspended in 250 μl of 1.12% sodium citrate buffer (pH 8.4) with 12.5 μg of RNase and incubated for an additional 30 min at 37°C. The cellular DNA was then stained by adding 250 μl of a propidium iodide solution (50 μg/ml) to the cells for 30 min at room temperature. The stained cells were analyzed by fluorescent-activated cell sorting on a FACScan flow cytometer (BD Biosciences, San Jose, CA) to determine the relative DNA content, which was based on the red fluorescence intensity.

### Western blot analysis

Cells were washed with cold PBS and lysed on ice in 50 μL of lysis buffer (50 mM Tris-HCl, 1 mM EGTA, 1% Triton X-100, 1 mM phenylmethylsulfonyl fluoride, pH 7.5). Lysates were centrifuged at 10,000 x g for 15 min at 4°C, and the supernatant fractions were collected. Proteins were separated by SDS-PAGE and transferred to an Immobilon-P membrane. Specific proteins were detected using an enhanced chemiluminescence (ECL) Western blot kit according to the manufacturer's instructions.

### 4′,6′-Diamidino-2-phenylindole staining (DAPI) for nuclei condensation and fragmentation

To examine cellular nuclei, the cells were fixed with 1% paraformaldehyde on glass slides for 30 min at room temperature. After the fixation, the cells were washed with PBS and a 300 nM 4′,6′-diamidino-2-phenylindole solution (Roche, Basel, Switzerland) was added to the fixed cells for 5 min. After the nuclei were stained, the cells were examined by fluorescence microscopy (Carl Zeiss, Jena, Germany).

### Cell death assessment by DNA fragmentation assay

The cell death detection ELISA plus kit (Boehringer Mannheim, Indianapolis, IN) was used for assessing apoptotic activity by detecting fragmented DNA within the nucleus in YM155-, TRAIL-, and combination with YM155 and TRAIL-treated cells. Briefly, each culture plate was centrifuged for 10 min at 200 x g, the supernatant was removed, and the pellet was lysed for 30 min. After centrifuging the plate again at 200 x g for 10 min, and the supernatant that contained the cytoplasmic histone-associated DNA fragments was collected and incubated with an immobilized anti-histone antibody. The reaction products were incubated with a peroxidase substrate for 5 min and measured by spectrophotometry at 405 and 490 nm (reference wavelength) with a microplate reader. The signals in the wells containing the substrate alone were subtracted as the background.

### Asp-Glu-Val-Asp-ase (DEVDase) activity assay

To evaluate DEVDase activity, cell lysates were prepared after their respective treatments with YM155 in the presence or absence of TRAIL. Assays were performed in 96-well microtiter plates by incubating 20 mg of cell lysates in 100 ml of reaction buffer (1% NP-40, 20 mM Tris-HCl, pH 7.5, 137 mM NaCl, 10% glycerol) containing a caspase substrate [Asp-Glu-Val-Asp-chromophore-p-nitroanilide (DVAD-pNA)] at 5 mM. Lysates were incubated at 37°C for 2 h. Thereafter, the absorbance at 405 nm was measured with a spectrophotometer.

### Determination of synergy

The possible synergistic effect of YM155 and TRAIL was evaluated using the isobologram method. In brief, cells were treated with different concentrations of YM155 and TRAIL alone or in combination. After 24 h, relative survival was assessed, and the concentration effect curves were used to determine the IC50 (the half-maximal inhibitory concentration) values for each drug alone and in combination with a fixed concentration of the second agent.

### Clonogenic survival assay

Cells (0.5×10^5^) were seeded in a 12-well culture plates, and followed by treatment with YM155 in the presence or absence of TRAIL for 24 h, and then washed with PBS. After 3d, clonogenic survival was determined by staining colonies using 0.4% coomassie blue, and visualized by a digital camera.

### Determination for the mitochondrial membrane potential by rhodamine 123

Rhodamine 123 (Molecular Probes Inc., Eugene, OR) uptake by mitochondria is directly proportional to its membrane potential. After treatment, cells were incubated with rhodamine 123 (5 μM) for 5 min in the dark at 37°C. The cells were harvested and suspended in PBS. The mitochondrial membrane potential was subsequently analyzed using a flow cytometer.

### Analysis of cytochrome *c* release

The cells were harvested, washed once with ice-cold PBS and gently lysed for 2 min in 80 μl ice-cold lysis buffer [250 mM sucrose, 1 mM EDTA, 20 mM Tris–HCl (pH 7.2), 1 mM DTT, 10 mM KCl, 1.5 mM MgCl_2_, 5 μg/ml pepstatin A, 10 μg/ml leupeptin and 2 μg/ml aprotinin]. Lysates were centrifuged at 12,000 x g at 4°C for 10 min to obtain the supernatants (cytosolic extracts free of mitochondria) and the pellets (fraction that contains mitochondria). The resulting cytosolic fractions were used for Western blot analysis with an anti-cytochrome *c* antibody.

### Reverse transcription polymerase chain reaction (RT-PCR) and quantitative PCR (qPCR)

Total RNA was isolated using the TriZol reagent (Life Technologies; Gaithersburg, MD), and the cDNA was prepared using M-MLV reverse transcriptase (Gibco-BRL; Gaithersburg, MD) according to the manufacturers' instructions [47]. The following primers were used for the amplification of human Mcl-1, survivin, c-FLIP and actin: Mcl-1 (forward) 5′- GCG ACT GGC AAA GCT TGG CCT CAA -3′ and (reverse) 5′- GTT ACA GCT TGG ATC CCA ACT GCA -3′; survivin (forward) 5′- GGA CCA CCG CAT CTC TAC AT -3′and (reverse) 5′- GCA CTT TCT TCG CAG TTT CC -3′; c-FLIP (forward) 5′- CGG ACT ATA GAG TGC TGA TGG -3′ and (reverse) 5′- GAT TAT CAG GCA GAT TCC TAG -3′; and actin (forward) 5′- GGC ATC GTC ACC AAC TGG GAC -3′ and (reverse) 5′- CGA TTT CCC GCT CGG CCG TGG -3′. The PCR amplification was carried out using the following cycling conditions: 94°C for 3 min followed by 17 (actin) or 28 cycles (Mcl-1, survivin, and c-FLIP) of 94°C for 40 s, 56°C for 40 s, 72°C for 1 min, and a final extension at 72°C for 5 min. The amplified products were separated by electrophoresis on a 1.5% agarose gel and detected under UV light. For qPCR, cDNA and forward/reverse primers (200 nM) were added to 2 × KAPA SYBR Fast master mix, and reactions were performed on LightCycler 480 real-time amplification instrument (Roche, Basel, Switzerland). The following primers were used for the amplification of human Mcl-1, survivin, c-FLIP and actin: Mcl-1 (forward) 5′- ATG CTT CGG AAA CTG GAC AT -3′ and (reverse) 5′- TCC TGA TGC CAC CTT CTA GG -3′; survivin (forward) 5′- TTC TCA AGG ACC ACC GCA TC -3′ and (reverse) 5′- GTT TCC TTT GCA TGG GGT CG -3′; c-FLIP (forward) 5′- CGC TCA ACA AGA ACC AGT G -3′ and (reverse) 5′- AGG GAA GTG AAG GTG TCT C -3′ and actin (forward) 5′- CTA CAA TGA GCT GCG TGT G-3′ and (reverse) 5′-TGG GGT GTT GAA GGT CTC -3′. Threshold cycle number (Ct) of each gene was calculated, and actin was used as reference genes. Delta-delta Ct values of genes were presented as relative fold induction [48].

### Stable transfection in Caki cell

The Caki cells were transfected in a stable manner with the pFLAG-CMV4/Mcl-1 plasmid, pcDNA3.1/survivin-flag plasmid, pcDNA 3.1/c-FLIP plasmid or control plasmid pcDNA 3.1 vector using Lipofectamine2000 as prescribed by the manufacturer (Invitrogen, Carlsbad, CA). After 48 h of incubation, transfected cells were selected in primary cell culture medium containing 700 μg/mL G418 (Invitrogen, Carlsbad, CA). After 2 or 3 weeks, single independent clones were randomly isolated, and each individual clone was plated separately. After clonal expansion, cells from each independent clone were tested for Mcl-1, survivin and c-FLIP expression by immunoblotting.

### Small interfering RNA (siRNA)

The cathepsin S siRNA used in this study was purchased from Santa Cruz Biotechnology (Dallas, TX). The green fluorescent protein (GFP) (control) siRNA was purchased from Bioneer (Daejeon, Korea). Cells were transfected with siRNA oligonucleotides using Lipofectamine® RNAiMAX Reagent (Invitrogen, Carlsbad, CA) according to the manufacturer's recommendations.

### Measurement of lysosomal membrane permeabilization

Lysotracker Red (Molecular Probes Inc., Eugene, OR) uptake by lysosome is proportional to its membrane potential. Caki cells were treated with 50 nM YM155 for the indicated times periods, then cells were incubated with 2.5 μM lysotracker red for 5 min at 37°C. The cells were then trypsinized and resuspended in PBS, and fluorescence was measured at specific time intervals with a flow cytometer.

### Fractionation of cytosol and membrane extracts

Cells were washed with ice-cold PBS, then resuspended in cytosol extraction buffer (250 mM sucrose, 10 mM KCl, 1.5 mM MgCl_2_, 1 mM EDTA, 1 mM EGTA, 20 mM HEPES) containing a 250 μg/ml digitonin and left on ice for 10 min and then lysate was centrifuged at 13,000 x g for 90 seconds. The supernatant (cytosol) was transferred to a new tube and pellets (membrane fraction) were suspended with lysis buffer. Lysates were centrifuged at 13,000 x g at 4°C for 15 min to obtain the supernatant fractions were collected as the membrane extract.

### Cathepsin S enzyme activity assay

Cathepsin S activity assay kit (Fluorometric) was purchased from Abcam (Cambridge, MA). The cells were collected and lysed on ice in cathepsin S cell lysis buffer for 10 min. The lysates were centrifuged at 13,000 x g for 5 min at 4°C, and the supernatant fractions were collected. Assay were performed in 96-well microtiter plates by incubation 50 μg of cell lysates in 50 μl of reaction buffer containing a 200 μM cathepsin S substrate (Ac-VVR-AFC). Lysates were incubated at 37°C for 1 h. The enzyme activity was measured with a fluorometric plate reader at an excitation wavelength of 400 nm and an emission wavelength of 505 nm. Fold-increase in cathepsin S activity was determined by comparing the relative fluorescence units (RFU) with the level of the control sample.

### DNA transfection and luciferase assay

Transient transfection was performed in 6-well plates. One day before the transfection, Caki cells were plated at approximately 60 to 80% confluence. The NF-κB, AP-1 and Sp1 promoter plasmid was transfected into the cells using Lipofectamine^TM^ 2000 (Invitrogen, Carlsbad, CA). To assess the promoter-driven expression of the luciferase gene, the cells were collected and disrupted by sonication in lysis buffer (25 mM Tris-phosphate, pH 7.8, 2 mM EDTA, 1% Triton X-100, and 10% glycerol), and aliquots of the supernatant were used to analyze the luciferase activity according to the manufacturer's instructions (Promega, Madison, WI).

### Statistical analysis

The data were analyzed using a one-way ANOVA and post-hoc comparisons (Student-Newman-Keuls) using the Statistical Package for Social Sciences 22.0 software (SPSS Inc., Chicago, IL).

## SUPPLEMENTARY FIGURES


