# Supplementary material for: YM155 sensitizes TRAIL-induced apoptosis through cathepsin S-dependent down-regulation of Mcl-1 and NF-κB-mediated down-regulation of c-FLIP expression in human renal carcinoma Caki cells
Source: Oncotarget. 2016 Aug 9;7(38):61520–32. doi: 10.18632/oncotarget.11137 (PMC5308669; doi:10.18632/oncotarget.11137)
Supplement: Supplementary file 1 [file oncotarget-07-61520-s001.pdf]

# YM155 sensitizes TRAIL-induced apoptosis through cathepsin S-dependent down-regulation of Mcl-1 and NF-κB-mediated down-regulation of c-FLIP expression in human renal carcinoma Caki cells

## SUPPLEMENTARY FIGURES

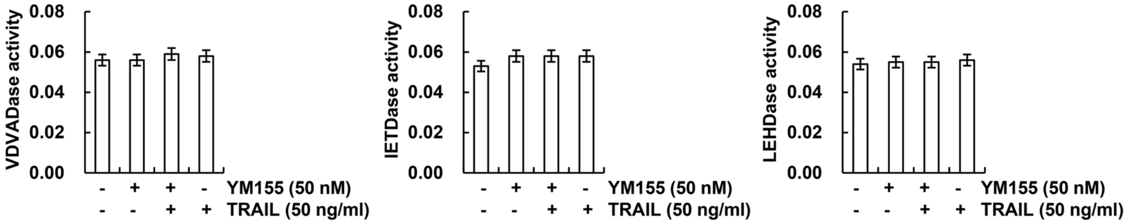


**Supplementary Figure S1: Effect of combined treatment with YM155 and TRAIL on various caspases activity.** Caki cells were treated with 50 ng/ml TRAIL in the presence or absence of 50 nM YM155 for 24 h. Caspase activities were determined with colorimetric assays using caspase-2 (VDVADase), caspase-8 (IETDase) and caspase-9 (LEHDase) assay kits. The values in figure represent the mean ± SD from three independent samples.

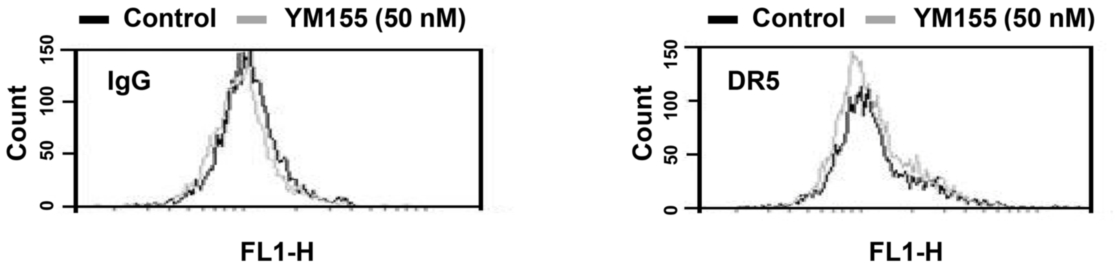


**Supplementary Figure S2: Effect of YM155 in DR5 expression on the cell surface.** Caki cells were treated with 50 nM YM155 for 24 h. Caki cells were labeled with control IgG and anti-DR5 antibody, and incubated with secondary FITC-conjugated antibody. The cell surface expression levels of DR5 were measured by flow cytometry.

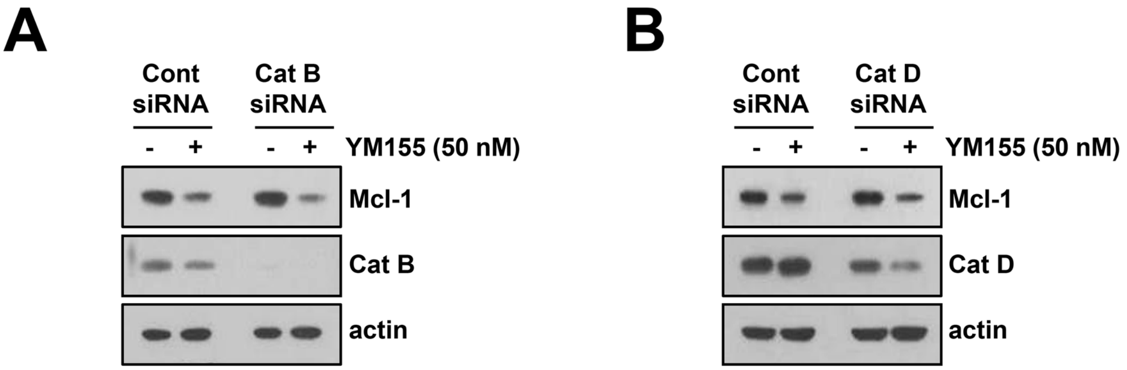


**Supplementary Figure S3: Knock-down of cathepsin B or cathepsin D by siRNA had no effect on YM155-induced down-regulation of Mcl-1 expression. A** and **B.** Caki cells were transiently transfected control siRNA (Cont siRNA), cathepsin B siRNA (Cat B siRNA, A) or cathepsin D siRNA (Cat D siRNA, B). Twenty-four hours after transfection, cells were treated with 50 nM YM155 for 24 h. The protein expression levels of Mcl-1, cathepsin B, cathepin D and actin were determined by western blotting. The level of actin was used as a loading control.

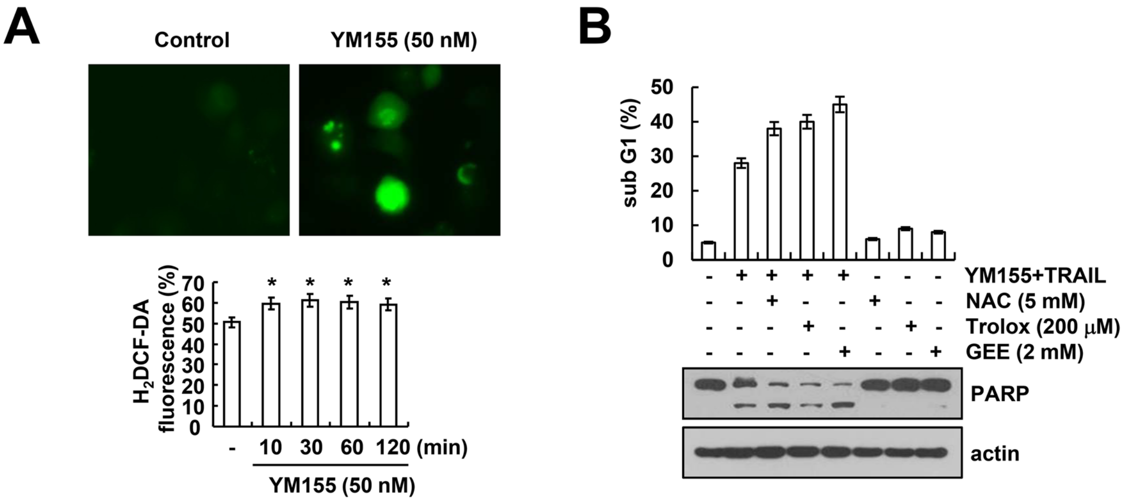


**Supplementary Figure S4: YM155 and TRAIL-induced apoptosis is independent of ROS signaling in Caki cells. A.** Caki cells were treated with 50 nM YM155 for 30 min and loaded with a $H_2$DCFDA fluorescent dye. The fluorescence intensity was detected by fluorescence microscopy (upper panel) and flow cytometry (lower panel). **B.** Caki cells were pretreated with 5 mM NAC, 200 μM trolox and 2 mM GEE for 30 min, and then treated with 50 nM YM155 plus 50 ng/ml TRAIL for 24 h. The level of apoptosis was analyzed by the sub-G1 fraction using flow cytometry (upper panel). The protein expression levels of PARP and actin were determined by western blotting. The level of actin was used as a loading control (lower panel). The values in panel (A and B) represent the mean ± SD from three independent samples. * $p < 0.05$ compared to the control.
